# Tetra-μ-acetato-bis­[(1,3-benzothia­zole)copper(II)](*Cu*—*Cu*)

**DOI:** 10.1107/S1600536811027140

**Published:** 2011-07-13

**Authors:** Johannes Hermle, Gerd Meyer

**Affiliations:** a Department für Chemie, Institut für Anorganische Chemie, Universität zu Köln, Greinstrasse 6, 50939 Köln, Germany

## Abstract

The title compound, [Cu_2_(CH_3_CO_2_)_4_(C_7_H_5_NS)_2_] or [(C_7_H_5_NS)Cu]_2_(μ-O_2_CCH_3_)_4_, crystallizes with one mol­ecule per unit cell. The coordination number of copper is six with four basal O atoms, one axial N atom and one axial Cu atom. Four acetate ligands act as bidentate linker and connect two Cu atoms, with a crystallographic inversion center located at the mid-point of the Cu—Cu bond. The acetate ligands form slightly distorted square planes around each metal ion, while the copper ions are displaced by 0.2089 (4) Å from these planes towards the N atoms. Thus, the Cu—Cu distance is elongated to 2.6378 (7) Å, compared with the 2.2180 (7) Å distance between the two basal planes. The angle between the basal plane and the Cu—N bond is 4.84 (6)°.

## Related literature

The structural prototype of (*L*Cu)_2_(*μ*-O_2_CCH_3_)_4_ complexes is the crystal structure of cupric acetate monohydrate (*L* = water), see: Van Niekerk & Schoening (1953 ▶); Ferguson & Glidewell (2003 ▶). For similar structures with *L* = benzimidazole, see: Bukowska-Strzyżewska *et al.* (1982) ▶ and *L* = 2-amino-benzothia­zole, see: Sun *et al.* (2007 ▶). For theoretical studies see: Rodríguez-Fortea *et al.* (2001) ▶ and for magnetic properties of dinuclear copper complexes, see: Tokii & Muto (1983 ▶). For FIR spectroscopic data and the magnetic moment of the complex with *L* = benzothia­zole, see: Ford *et al.* (1968 ▶).
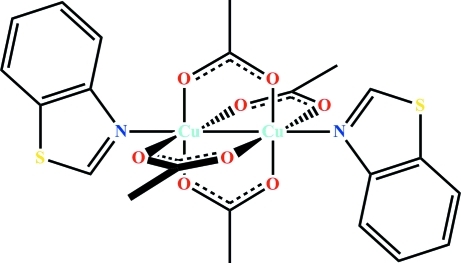


## Experimental

### 

#### Crystal data

[Cu_2_(C_2_H_3_O_2_)_4_(C_7_H_5_NS)_2_]
*M* *_r_* = 633.66
Triclinic, 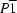

*a* = 7.185 (1) Å
*b* = 8.1918 (12) Å
*c* = 11.8265 (16) Å
α = 106.516 (16)°
β = 106.429 (16)°
γ = 97.344 (17)°
*V* = 623.49 (18) Å^3^
*Z* = 1
Mo *K*α radiation
μ = 1.92 mm^−1^
*T* = 293 K
0.3 × 0.2 × 0.1 mm

#### Data collection

Stoe IPDS I diffractometer
Absorption correction: numerical (*X-SHAPE*; Stoe & Cie, 1999 ▶) *T*_min_ = 0.575, *T*_max_ = 0.840
7525 measured reflections
2784 independent reflections
2092 reflections with *I* > 2σ(*I*)
*R*_int_ = 0.038

#### Refinement

*R*[*F* ^2^ > 2σ(*F* ^2^)] = 0.032
*wR*(*F* ^2^) = 0.075
*S* = 0.97
2784 reflections
165 parameters
H-atom parameters constrained
Δρ_max_ = 0.45 e Å^−3^
Δρ_min_ = −0.45 e Å^−3^

### 

Data collection: *X-AREA* (Stoe & Cie, 2001 ▶); cell refinement: *X-AREA*; data reduction: *X-AREA*; program(s) used to solve structure: *SIR92* (Altomare *et al.*, 1993 ▶); program(s) used to refine structure: *SHELXL97* (Sheldrick, 2008 ▶); molecular graphics: *DIAMOND* (Brandenburg, 2011 ▶); software used to prepare material for publication: *WinGX* (Farrugia, 1999 ▶).

## Supplementary Material

Crystal structure: contains datablock(s) I, global. DOI: 10.1107/S1600536811027140/hg5042sup1.cif

Structure factors: contains datablock(s) I. DOI: 10.1107/S1600536811027140/hg5042Isup2.hkl

Additional supplementary materials:  crystallographic information; 3D view; checkCIF report

## Figures and Tables

**Fig. 1.**
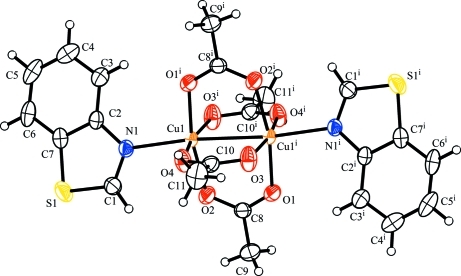
The molecular structure of (I), with atom labels and 50% probability displacement ellipsoids for non-H atoms. [Symmetry code: (i) -x + 1, -y + 1, -z + 1]

**Fig. 2.**
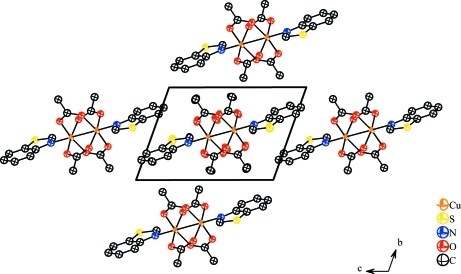
The packing of (I), viewed along the b axis, H atoms have been omitted for clarity.

**Fig. 3.**
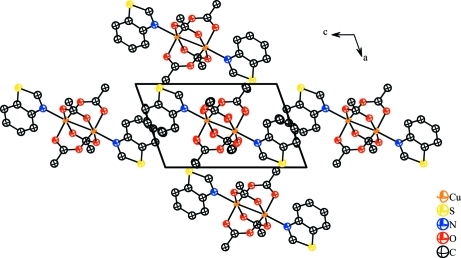
The packing of (I), viewed along the a axis, H atoms have been omitted for clarity.

## supplementary crystallographic information

### Comment

Copper(II) acetate complexes of the general formula [LCu]_2_(*µ*^2^-OAc)_4_, where *L* is a ligand with an oxygen or nitrogen ligator atom have been well explored. The structural evidence of a copper-copper bond was given by van Niekerk & Schoening in 1953. The title compound is a dinuclear complex disposed around an inversion center located at the mid-point of the Cu—Cu bond. The coordination environment of Cu(II) ions can be described as a slightly distorted octahedron, with each copper atom being surrounded by four *µ*^2^-bridging bidentate acetate ligands in the basal plane and one benzothiazole ligand in one axial position (Fig. 1). The sixth coordination site is occupied by the neighbouring copper(II) atom. The Cu—Cu distance is about 0.02 Å longer as in Cu(II) acetate monohydrate [(H_2_O)Cu]_2_(µ^2^-OAc)_4_ with 2.6157 (8) Å (Ferguson & Glidewell 2003) and 0.03 Å shorter than in the benzimidazole complex 2.663 (1) Å (Bukowska-Strzyzewska *et al.* 1982).  To our knowledge there is only one more dinuclear complex with acetate ligands and a benzothiazole derivative known (Sun *et al.* 2007). The magnetic moment of [(C_7_H_5_NS)Cu]_2_(*µ*^2^-OAc)_4_ of *µ* = 1.42 *µ*_B_ at room temperature is an evidence for Cu—Cu interactions with coupling of the electron spins, Ford *et al.* 1968. Magnetic susceptibilities of benzothiazole, thiazole and thiazole derivatives have been measured by Tokii & Muto 1983. Theoretical studies on intramolecular antiferromagnetic coupling in carboxylato-bridged dinuclear copper(II) complexes have been performed by Rodríguez-Fortea *et al.* 2001.

### Experimental

Green platelets of [(C_7_H_5_NS)Cu]_2_(*µ*^2^-OAc)_4_ were obtained by the reaction of 0.20 g cupric acetate monohydrate (1 mmol, Merck) with 0.22 ml benzothiazole (0.27 g, 2 mmol, Acros) in 20 ml of ethanol at ambient temperature by slow evaporation of the solvent within two weeks. Yield: 0.59 g (93%). Mp: 206 °C (Decomp.). UV/VIS: (chloroform) λ_max_ 358, 690 nm. IR: 3080(w), 3057(w), 2995(w), 2927(w), 1612(*s*), 1562(*m*), 1468(*m*), 1454(*m*), 1431(*s*), 1348(*m*), 1321(w), 1304(*m*), 1273(w), 1205(w), 1155(w), 1066(w), 1049(w), 1030(w), 1016(w), 949(w), 897(*m*), 870(w), 856(w), 810(w), 762(*m*), 733(*m*), 681(*m*), 627(*m*), 534(w), 507(w), 424(w) cm^-1^. Elem. Anal. calcd for C_22_H_22_Cu_2_N_2_O_8_S_2_: C, 41.70; H, 3.50; N, 4.42; S, 10.12; found: C, 41.30; H, 3.30; N, 4.17; S, 10.14.

### Refinement

Hydrogen atoms were placed in idealized positions and constrained riding on their parent atoms [C–H = 0.93–0.96 Å with *U*_iso_(H) = 1.2 *U*_eq_(C)]. The last cycles of refinement included atomic positions for all atoms, anisotropic thermal parameters for all non-hydrogen atoms and isotropic thermal parameters for all hydrogen atoms.

### Crystal data

**Table:**

[Cu_2_(C_2_H_3_O_2_)_4_(C_7_H_5_NS)_2_]	*Z* = 1
*M**_r_* = 633.66	*F*(000) = 322
Triclinic, *P*1	*D*_x_ = 1.688 Mg m^−^^3^
Hall symbol: -P 1	Mo *K*α radiation, λ = 0.71073 Å
*a* = 7.185 (1) Å	Cell parameters from 1512 reflections
*b* = 8.1918 (12) Å	θ = 3.8–56.3°
*c* = 11.8265 (16) Å	µ = 1.92 mm^−^^1^
α = 106.516 (16)°	*T* = 293 K
β = 106.429 (16)°	Plate, green
γ = 97.344 (17)°	0.3 × 0.2 × 0.1 mm
*V* = 623.49 (18) Å^3^	

### Data collection

**Table:**

Stoe IPDS I diffractometer	2784 independent reflections
Radiation source: fine-focus sealed tube	2092 reflections with *I* > 2σ(*I*)
graphite	*R*_int_ = 0.038
Detector resolution: 0 pixels mm^-1^	θ_max_ = 28.1°, θ_min_ = 2.7°
Oscillation scans	*h* = −8→8
Absorption correction: numerical (*X-SHAPE*; Stoe & Cie, 1999)	*k* = −10→10
*T*_min_ = 0.575, *T*_max_ = 0.840	*l* = −15→15
7525 measured reflections	

### Refinement

**Table:**

Refinement on *F*^2^	Primary atom site location: structure-invariant direct methods
Least-squares matrix: full	Secondary atom site location: difference Fourier map
*R*[*F*^2^ > 2σ(*F*^2^)] = 0.032	Hydrogen site location: inferred from neighbouring sites
*wR*(*F*^2^) = 0.075	H-atom parameters constrained
*S* = 0.97	*w* = 1/[σ^2^(*F*_o_^2^) + (0.0379*P*)^2^] where *P* = (*F*_o_^2^ + 2*F*_c_^2^)/3
2784 reflections	(Δ/σ)_max_ = 0.013
165 parameters	Δρ_max_ = 0.45 e Å^−^^3^
0 restraints	Δρ_min_ = −0.45 e Å^−^^3^

### Special details

**Table:**

Experimental. A single crystal suitable for X-ray diffraction was selected under a polarization microscope and sealed in a capillary tube. Complete scattering intensities data sets were collected with an imaging plate diffractometer (*IPDS* I, Stoe & Cie). The data were corrected for Lorentz and polarization effects. A numerical absorption correction based on crystal-shape optimization was applied for all data.
Geometry. All e.s.d.'s (except the e.s.d. in the dihedral angle between two l.s. planes) are estimated using the full covariance matrix. The cell e.s.d.'s are taken into account individually in the estimation of e.s.d.'s in distances, angles and torsion angles; correlations between e.s.d.'s in cell parameters are only used when they are defined by crystal symmetry. An approximate (isotropic) treatment of cell e.s.d.'s is used for estimating e.s.d.'s involving l.s. planes.
Refinement. Refinement of *F*^2^ against ALL reflections. The weighted *R*-factor *wR* and goodness of fit *S* are based on *F*^2^, conventional *R*-factors *R* are based on *F*, with *F* set to zero for negative *F*^2^. The threshold expression of *F*^2^ > σ(*F*^2^) is used only for calculating *R*-factors(gt) *etc*. and is not relevant to the choice of reflections for refinement. *R*-factors based on *F*^2^ are statistically about twice as large as those based on *F*, and *R*- factors based on ALL data will be even larger. Hydrogen atoms were placed in idealized positions and constrained riding on their parent atoms [C–H = 0.93–0.96 Å with *U*_iso_(H) = 1.2 *U*_eq_(C)]. The last cycles of refinement included atomic positions for all atoms, anisotropic thermal parameters for all non-hydrogen atoms and isotropic thermal parameters for all hydrogen atoms.

### Fractional atomic coordinates and isotropic or equivalent isotropic displacement parameters (Å^2^)

**Table:**

	*x*	*y*	*z*	*U*_iso_*/*U*_eq_	
Cu1	0.43471 (5)	0.46234 (4)	0.58527 (3)	0.02316 (11)	
S1	0.04719 (13)	0.43274 (11)	0.83857 (7)	0.0430 (2)	
N1	0.2932 (4)	0.4019 (3)	0.71570 (19)	0.0265 (5)	
O3	0.6747 (3)	0.6371 (3)	0.70893 (18)	0.0428 (5)	
O4	0.7826 (3)	0.7015 (3)	0.56499 (17)	0.0390 (5)	
O1	0.5911 (3)	0.2831 (3)	0.56937 (19)	0.0374 (5)	
C2	0.3612 (4)	0.3329 (3)	0.8112 (2)	0.0259 (6)	
O2	0.6990 (4)	0.3443 (3)	0.4247 (2)	0.0427 (6)	
C6	0.3008 (5)	0.2858 (4)	0.9930 (3)	0.0411 (8)	
H6	0.2271	0.2953	1.0468	0.049*	
C4	0.5758 (5)	0.2012 (4)	0.9319 (3)	0.0458 (8)	
H4	0.6848	0.1503	0.9462	0.055*	
C5	0.4659 (6)	0.2169 (4)	1.0127 (3)	0.0462 (9)	
H5	0.5055	0.1798	1.0813	0.055*	
C11	0.9534 (6)	0.8715 (4)	0.7759 (3)	0.0542 (10)	
H11A	0.9499	0.8716	0.8564	0.081*	
H11B	0.9337	0.9812	0.7663	0.081*	
H11C	1.0803	0.8550	0.7693	0.081*	
C9	0.7821 (5)	0.1025 (4)	0.4827 (3)	0.0415 (7)	
H9A	0.9197	0.1415	0.4931	0.062*	
H9C	0.7166	0.0146	0.4018	0.062*	
H9B	0.7733	0.0542	0.5466	0.062*	
C1	0.1335 (5)	0.4564 (4)	0.7205 (3)	0.0344 (7)	
H1	0.0685	0.5058	0.6633	0.041*	
C3	0.5264 (5)	0.2595 (4)	0.8310 (3)	0.0359 (7)	
H3	0.6015	0.2501	0.7780	0.043*	
C7	0.2462 (5)	0.3417 (3)	0.8892 (2)	0.0307 (6)	
C10	0.7917 (4)	0.7261 (3)	0.6755 (2)	0.0288 (6)	
C8	0.6835 (4)	0.2542 (3)	0.4930 (2)	0.0278 (6)	

### Atomic displacement parameters (Å^2^)

**Table:**

	*U*^11^	*U*^22^	*U*^33^	*U*^12^	*U*^13^	*U*^23^
Cu1	0.0256 (2)	0.02769 (17)	0.02156 (16)	0.00738 (12)	0.01074 (12)	0.01282 (12)
S1	0.0358 (5)	0.0654 (5)	0.0383 (4)	0.0139 (4)	0.0222 (4)	0.0223 (4)
N1	0.0267 (14)	0.0329 (12)	0.0228 (11)	0.0055 (9)	0.0097 (9)	0.0127 (9)
O3	0.0453 (15)	0.0499 (12)	0.0239 (10)	−0.0096 (10)	0.0076 (9)	0.0115 (9)
O4	0.0353 (13)	0.0522 (13)	0.0247 (10)	−0.0037 (10)	0.0095 (9)	0.0124 (9)
O1	0.0467 (14)	0.0429 (11)	0.0437 (12)	0.0252 (10)	0.0268 (10)	0.0275 (10)
C2	0.0283 (16)	0.0263 (12)	0.0203 (12)	−0.0001 (10)	0.0065 (10)	0.0082 (10)
O2	0.0588 (16)	0.0442 (12)	0.0552 (13)	0.0307 (11)	0.0389 (12)	0.0338 (11)
C6	0.061 (2)	0.0362 (16)	0.0259 (14)	−0.0033 (14)	0.0177 (14)	0.0124 (13)
C4	0.056 (2)	0.0381 (16)	0.0425 (18)	0.0160 (15)	0.0071 (16)	0.0191 (15)
C5	0.073 (3)	0.0337 (16)	0.0285 (15)	0.0051 (15)	0.0081 (15)	0.0182 (13)
C11	0.055 (2)	0.050 (2)	0.0364 (18)	−0.0127 (16)	0.0009 (16)	0.0081 (15)
C9	0.045 (2)	0.0338 (15)	0.056 (2)	0.0195 (14)	0.0224 (16)	0.0201 (15)
C1	0.0300 (18)	0.0488 (17)	0.0292 (14)	0.0109 (13)	0.0104 (12)	0.0188 (13)
C3	0.040 (2)	0.0374 (15)	0.0337 (15)	0.0099 (13)	0.0121 (13)	0.0175 (13)
C7	0.0354 (18)	0.0305 (14)	0.0236 (13)	−0.0010 (11)	0.0114 (11)	0.0074 (11)
C10	0.0269 (17)	0.0295 (14)	0.0261 (13)	0.0053 (11)	0.0031 (11)	0.0102 (11)
C8	0.0253 (16)	0.0263 (13)	0.0327 (14)	0.0073 (11)	0.0087 (11)	0.0115 (11)

### Geometric parameters (Å, °)

**Table:**

Cu1—O1	1.9589 (19)	C6—C5	1.367 (5)
Cu1—O2^i^	1.9689 (19)	C6—C7	1.401 (3)
Cu1—O3	1.978 (2)	C6—H6	0.9300
Cu1—O4^i^	1.984 (2)	C4—C3	1.381 (4)
Cu1—N1	2.203 (2)	C4—C5	1.391 (5)
Cu1—Cu1^i^	2.6378 (7)	C4—H4	0.9300
S1—C1	1.727 (3)	C5—H5	0.9300
S1—C7	1.732 (3)	C11—C10	1.497 (4)
N1—C1	1.293 (4)	C11—H11A	0.9600
N1—C2	1.398 (3)	C11—H11B	0.9600
O3—C10	1.261 (3)	C11—H11C	0.9600
O4—C10	1.246 (3)	C9—C8	1.500 (4)
O4—Cu1^i^	1.984 (2)	C9—H9A	0.9600
O1—C8	1.253 (3)	C9—H9C	0.9600
C2—C3	1.390 (4)	C9—H9B	0.9600
C2—C7	1.396 (4)	C1—H1	0.9300
O2—C8	1.256 (3)	C3—H3	0.9300
O2—Cu1^i^	1.9689 (19)		
			
O1—Cu1—O2^i^	167.71 (7)	C5—C4—H4	119.3
O1—Cu1—O3	90.12 (10)	C6—C5—C4	121.3 (2)
O2^i^—Cu1—O3	88.37 (10)	C6—C5—H5	119.4
O1—Cu1—O4^i^	88.25 (10)	C4—C5—H5	119.4
O2^i^—Cu1—O4^i^	90.67 (10)	C10—C11—H11A	109.5
O3—Cu1—O4^i^	167.89 (8)	C10—C11—H11B	109.5
O1—Cu1—N1	100.05 (8)	H11A—C11—H11B	109.5
O2^i^—Cu1—N1	92.24 (8)	C10—C11—H11C	109.5
O3—Cu1—N1	98.77 (8)	H11A—C11—H11C	109.5
O4^i^—Cu1—N1	93.33 (8)	H11B—C11—H11C	109.5
O1—Cu1—Cu1^i^	84.48 (6)	C8—C9—H9A	109.5
O2^i^—Cu1—Cu1^i^	83.24 (6)	C8—C9—H9C	109.5
O3—Cu1—Cu1^i^	85.57 (6)	H9A—C9—H9C	109.5
O4^i^—Cu1—Cu1^i^	82.33 (6)	C8—C9—H9B	109.5
N1—Cu1—Cu1^i^	173.67 (6)	H9A—C9—H9B	109.5
C1—S1—C7	88.85 (13)	H9C—C9—H9B	109.5
C1—N1—C2	110.6 (2)	N1—C1—S1	116.61 (19)
C1—N1—Cu1	118.25 (16)	N1—C1—H1	121.7
C2—N1—Cu1	130.51 (18)	S1—C1—H1	121.7
C10—O3—Cu1	121.50 (17)	C4—C3—C2	118.0 (3)
C10—O4—Cu1^i^	125.44 (19)	C4—C3—H3	121.0
C8—O1—Cu1	123.50 (15)	C2—C3—H3	121.0
C3—C2—C7	120.5 (2)	C2—C7—C6	120.8 (3)
C3—C2—N1	125.5 (2)	C2—C7—S1	109.93 (18)
C7—C2—N1	114.0 (2)	C6—C7—S1	129.2 (2)
C8—O2—Cu1^i^	124.42 (18)	O4—C10—O3	124.7 (3)
C5—C6—C7	117.9 (3)	O4—C10—C11	117.7 (3)
C5—C6—H6	121.0	O3—C10—C11	117.6 (2)
C7—C6—H6	121.0	O1—C8—O2	124.2 (2)
C3—C4—C5	121.4 (3)	O1—C8—C9	117.9 (2)
C3—C4—H4	119.3	O2—C8—C9	118.0 (3)

Symmetry codes: (i) −*x*+1, −*y*+1, −*z*+1.
